# Effects of four potent entomopathogenic fungal isolates on the survival and performance of *Telenomus remus*, an egg parasitoid of fall armyworm

**DOI:** 10.3389/fcimb.2024.1445156

**Published:** 2024-09-12

**Authors:** Junitor Chepkemoi, Ken Okwae Fening, Felicitas Chaba Ambele, Joseph Munywoki, Komivi Senyo Akutse

**Affiliations:** ^1^ International Centre of Insect Physiology and Ecology (icipe), Nairobi, Kenya; ^2^ African Regional Postgraduate Program in Insect Science (ARPPIS), University of Ghana, Legon, Accra, Ghana; ^3^ Department of Zoology, The University of Bamenda, Bambili, Cameroon; ^4^ Unit of Environmental Sciences and Management, North-West University, Potchefstroom, South Africa

**Keywords:** biological control, *Beauveria bassiana*, fall armyworm, *Metarhizium anisopliae*, *Telenomus remus*, insect-microbe interactions

## Abstract

Fall armyworm (FAW), *Spodoptera frugiperda* is a generalist pest known to feed on more than 300 plant species, including major staple crops such as rice, maize and sorghum. Biological control of FAW using a combination of a major indigenous egg parasitoid *Telenomus remus* and entomopathogenic fungi was explored in this study. *Metarhizium anisopliae* strains (ICIPE 7, ICIPE 41, and ICIPE 78) and *Beauveria bassiana* ICIPE 621 which demonstrated effectiveness to combat the pest, were evaluated through direct and indirect fungal infection to assess their pathogenicity and virulence against *T. remus* adults, *S. frugiperda* eggs and their effects on *T. remus* parasitism rates. *Metarhizium anisopliae* ICIPE 7 and ICIPE 78 exhibited the highest virulence against *T. remus* adults with LT_50_ values >2 days. ICIPE 7 induced the highest *T. remus* mortality rate (81.40 ± 4.17%) following direct infection with dry conidia. Direct fungal infection also had a significant impact on parasitoid emergence, with the highest emergence rate recorded in the *M. anisopliae* ICIPE 7 treatment (42.50 ± 5.55%), compared to the control ± (83.25 ± 5.94%). In the indirect infection, the highest concentration of 1 x 10^9^ conidia ml^-1^ of ICIPE 78 induced the highest mortality (100 ± 0.00%) of *T. remus* adults, and the highest mortality (51.25%) of FAW eggs, whereas the least FAW egg mortality (15.25%) was recorded in the lowest concentration 1 x 10^5^ conidia ml^-1^ of ICIPE 41. The number of parasitoids that emerged and their sex ratios were not affected by the different fungal strain concentrations except in ICIPE 7 at high dose. This study showed that potential combination of both *M. anisopliae* and *B. bassiana* with *T. remus* parasitoid can effectively suppress FAW populations.

## 1 Introduction

Fall armyworm (FAW), *Spodoptera frugiperda* J.E. Smith (Lepidoptera: Noctuidae) is a highly polyphagous pest that preferentially affects several agricultural crops such as maize, sorghum, and rice (Day et al., 2017). The pest spread from its native regions in America over the past few years, and currently an invasive species in Africa and East Asia countries (Hruska, 2019). Within sub-Saharan Africa, the pest was initially reported in the West African countries of Nigeria, Togo, and Benin in 2016, but has since spread to virtually all other countries (Goergen et al., 2016). FAW populations in Africa consist of both corn strain and rice strain and were estimated to cause maize yield losses ranging from 8.3 to 20.6 million tons per year according to a study conducted in 12 African maize-producing countries (Prasanna et al., 2018). These significant losses highly impact the diet of many residents in Africa because maize is reported to account for almost half of all calories and proteins consumed in Eastern and Southern Africa (Macauley, 2015).

Several methods for controlling FAW have been proposed but most have had little impact, with the efficacy of some solutions being questionable due to their inadequate assessment (Harrison et al., 2019). Although FAW has been the subject of extensive scientific research in its native range in America, chemical control methods continue to dominate its management, while the adoption of biological control strategies remain limited globally (Wyckhuys et al., 2024). Since the advent of FAW in African countries, insecticides have been commonly used as an emergency management approach to halt pest distribution and minimize damage in maize fields. Some of the pesticides used in the control of *S. frugiperda* include extremely dangerous methomyl, methyl parathion, endosulfan, and lindane (Bateman et al., 2018). In some parts of the world, certain pesticides, such as endosulfan and lindane, have been banned (Roşca et al., 2023). Biological control agents, such as fungal parasitoids and biopesticides are promising and sustainable alternatives. Therefore, appropriate and effective control strategies should be implemented in order to be able to combat this pest (Abbas et al., 2022).

The development and application of entomopathogens as biocontrol agents have led to several successes and have been commercialized to suppress insect pests. Over 50 entomopathogenic fungi, viruses, nematodes, and bacteria are currently commercialized and utilized as microbial biopesticides (Morales-Reyes et al., 2018). In the quest to design more sustainable and ecofriendly pest control strategies, some key potent fungal-based biopesticides have been identified as effective in managing both FAW eggs and neonates (Akutse et al., 2019), as well as the adults (Akutse et al., 2020a). The investigators observed that *Metarhizium anisopliae s.l.*, ICIPE 78, ICIPE 40 and ICIPE 20 were most effective than the other tested strains in decreasing egg hatchability, achieving reductions by 87, 83 and, 79.5%, respectively. Additionally, these fungal strains also caused significant mortality to the neonates, following treatment with fungus after emergence. Specifically, *M. anisopliae s.l.* strains, ICIPE 41 and ICIPE 7 were the most effective, with larval mortality rates of 96.49% and 93.66% respectively. ICIPE 78 and ICIPE 7 which have been already commercialized as Achieve^®^ and Tickoff^®^ against spider mites and ticks, respectively by Real IPM in partnership with International Centre of Insect Physiology and Ecology (*icipe*) (Akutse et al., 2020b), could therefore be used as a potential ovicidal biopesticide to suppress FAW population in Africa (Akutse et al., 2019). Another study also demonstrated that *B. bassiana s.l.* ICIPE 621 and *M. anisopliae s.l.* ICIPE 7 outperformed all the other strains by causing 100% mortality of the FAW adults (Akutse et al., 2020).

In addition, some parasitoids such as *Cotesia icipe*, *Chelonus curvimaculatus*, *Telenomus remus, Charops ater*, *Palexorista zonata*, *Trichogramma chilonis* and *Coccygidium luteum* were also locally were identified to be effective in managing FAW egg and larvae (Sisay et al., 2019). Therefore, assessing how these effective biopesticides interact with parasitoids is crucial for enhancing FAW control within an integrated pest management (IPM) framework in Africa. This study, therefore, focused on evaluating the non-target impacts of the identified potent commercial biopesticides (*M. anisopliae* ICIPE 7, ICIPE 78, ICIPE 41, and *B. bassiana* ICIPE 621) on FAW egg parasitoid *T. remus.*

## 2 Materials and methods

### 2.1 Fall armyworm rearing

A total of 450 *S. frugiperda* larvae were collected from maize farms in Homa Bay and Siaya counties (−0.61401° Latitude, 34.09095° Longitude; 1215 m a.s.l.) from April-July 2017. They were maintained in small cages (30 × 30 × 30 cm) under laboratory conditions with a temperature of 25 ± 2 °C, relative humidity of 70 ± 10% and a 12-hour light: 12-hour dark photoperiod. The larvae collected from the field were reared on a semisynthetic diet until they pupated (Akutse et al., 2020a).

The pupae (about 50 per cage) were kept in sleeved Perspex cages (40 × 40 × 45 cm) until the adults emerged. The moths were fed with 10% honey solution, absorbed in cotton wool balls. The colony was then maintained on maize seedlings for more than 10 generations prior to the bioassays. Female moths (about 50 pairs) were released for oviposition and potted maize plants introduced into the cages. After oviposition, the plants were transferred in a separate ventilated cage (50 × 50 × 60 cm) after 24 hours’ exposure to monitor egg hatchability. Three days after hatching, the infested plants were transferred to clean cages (50 × 50 × 60 cm) with lids covered in fine netting for ventilation. To absorb excess moisture, the cages were lined with a paper towel. Fresh maize leaves were fed to the larvae every day until they pupated. The pupae were carefully removed from the cages, using a fine camel hair brush and transferred to sleeved Perspex cages (40 × 40 × 45 cm) until the moths emerged.

### 2.2 *Telenomus remus* parasitoids rearing

A colony of *T. remus* was established from a group of 15 wasps (1:2 males: females) sourced from parasitized eggs of FAW collected during a field survey in Yatta (01.23044°S; 37.45789°E). They were kept in glass vials in the rearing room at *icipe*’s Animal Rearing and Quarantine Unit (ARQU), at a room temperature of 25 ± 1°C, 12-hour light:12-hour dark photoperiod, and 60-70% relative humidity (RH) (Chepkemoi et al., 2023). The parasitoids were reared on newly laid FAW egg masses, mounted onto rectangular manila cards (1 cm x 5 cm) with white glue and introduced to the wasps for parasitism. The exposure date was noted on the back of each card to track and estimate the expected emergence time of the parasitoids (Kibii, 2021). Additionally, a piece of plain paper (2 cm x 1 cm) coated with honey was placed inside the glass vial as nourishment for the emerged parasitoids. Glass vials holding the parasitoids were stored on the laboratory shelves. About 3-4 days after exposure, the eggs darkened, indicating that they had been parasitized. The parasitized egg cards were then transferred to a separate clean glass vial, where they were incubated at room temperature to monitor emergence of the parasitoids. The parasitoids emerged after a period of about 6-13 days. This process was repeated to maintain the colony, which continued for 16 generations before commencing the bioassays (Kibii, 2021).

### 2.3 Fungal strains culture and conidia viability assessment

The fungal strains used in this study were sourced from the *icipe*’s Arthropod Germplasm Centre. The origins and details of the fungal strains (three *Metarhizium* and one *Beauveria*) are outlined in **Table 1**. *Metarhizium anisopliae s.l.* ICIPE 7, ICIPE 78, and ICIPE 41 strains were cultured on Sabouraud dextrose agar (SDA), while *B. bassiana s.l.* ICIPE 621 was cultured on Potato dextrose agar (PDA) in Petri dishes (9 cm diameter). The inoculated plates were incubated in complete darkness at a temperature of 25 ± 2°C. After three weeks, the conidia were harvested from surface cultures and suspended in 10 ml distilled water with 0.05% Triton X‐100 in universal bottles (30 ml), each containing three glass beads. The suspension was vortexed for 5 min at about 700 rpm to break the conidial clumps and create a consistent, homogeneous mixture (Akutse et al., 2013). Conidial concentrations were quantified using a hemocytometer under a light microscope. Prior to the bioassays, the conidial suspensions were adjusted to a concentration of 1 × 10^9^ conidia ml^-1^, and other concentrations were achieved through serial dilution (Akutse et al., 2013). Conidia viability was determined by spread-plating 0.1 ml of 3 x 10^6^ conidia ml^-1^ onto 9 cm Petri dishes containing SDA or PDA medium. A sterile microscope cover slip (2 x 2 cm) was placed on top of the agar in each plate. Plates were sealed with parafilm and incubated in complete darkness at 25 ± 2°C and examined after 18 - 20 hours. The percentage germination was calculated by counting 100 randomly selected conidia on the area covered by each coverslip under the light microscope (400x magnification) (Oliveira et al., 2015). Conidia was considered to have germinated if the length of the germ tube was at least twice the diameter of the conidium (Oliveira et al., 2015). Each plate represented a replicate and each strain had four replicates and replicated over time (Chepkemoi et al., 2023).

**Table 1:** Identity of fungal strains screened against *Telenomus remus* for their virulence assessment under laboratory conditions.

Fungal species	Stains	Source	Location/Country	Year of isolation	Germination ± SE (%)
*Metarhizium anisopliae s.l.*	ICIPE 7	*Rhipicephalus appendiculatus*	Rusinga island (Kenya)	1996	93.0 ± 1.3a
	ICIPE 78	*Temnoschoita nigroplagiata*	Ungoe (Kenya)	1990	94.2 ± 1.7a
	ICIPE 41	Soil	Kenya	1990	87.3 ± 2.3b
*Beauveria bassiana s.l.*	ICIPE 621	Soil	Kericho (Kenya)	2008	91.2 ± 1.04ab

Means within a column followed by same letter are not significantly different by Student-Newman-Keuls (SKN) test (P < 0.05).

### 2.4 Direct effects of Metarhizium anisopliae and Beauveria bassiana strains on Telenomus remus

The virulence of four entomopathogenic fungal (EPF) isolates *M. anisopliae s.l.* strains (ICIPE 7, ICIPE 78, ICIPE 41) and *B. bassiana* ICIPE 621 *s.l* was evaluated against *T. remus* adults. For each fungal treatment, 20 1-day-old *T. remus* adults (ratio of 1:2/male: female) were infected with 1 g of dry conidia using velvet-coated plastic jars (150 × 80 mm) (Chepkemoi et al., 2023). Twenty (20) adult parasitoids (ratio of 1:2 - males: females) were exposed to the device for 3 min to collect spores. Control treatments were introduced to fungus-free velvet plastic jars. After being exposed for 3 min, the parasitoids that were contaminated were placed in clean 40 ml glass vials and fed with honey. Mortality was recorded daily for 7 days by counting the dead parasitoids. Mycosis in the dead insects was evaluated, with the presence of conidia and hyphae on the cadavers confirming that mortality was due to fungal infection. A completely randomized design was employed to arrange all treatments with each treatment, including the controls, replicated four times over time (Opisa et al., 2018).

Twenty adult parasitoids (ratio of 1:2 - males: females) were exposed to 50 freshly (1-day old) laid FAW eggs for 24 hours after the parasitoids had been contaminated with the fungus. The eggs were then transferred to separate glass vials and monitored until the adult parasitoids emerged. FAW egg mortality and emergence of adult parasitoids were recorded. Additionally, emergence rates of both FAW and adult parasitoids, parasitism rates and sex ratio of the parasitoids were documented. Adult parasitoids were kept in separate vials and fed with honey. The mortality, fecundity, oviposition/parasitism rate, and egg hatchability of the infected parasitoids were also evaluated. The experiment was replicated four times under a completely randomized design (Chepkemoi et al., 2023).

### 2.5 Indirect effects of Metarhizium anisopliae and Beauveria bassiana strains on Telenomus remus

A hundred freshly laid eggs (1-day old) of *S. frugiperda* were sprayed directly with 10 ml at different concentrations (1.0 x 10^5^, 1.0 x 10^6^, 1.0 x 10^7^, 1.0 x 10^8^ and 1.0 x 10^9^ conidia ml^-1^) of *M. anisopliae* strains (ICIPE 7, ICIPE 78, ICIPE 41) and *B. bassiana* ICIPE 621 using the Burgerjon’s (1956) spray tower, before being transferred to potted maize plants (Chepkemoi et al., 2023). Control treatments were sprayed with sterile distilled water containing 0.05% Triton X-100. After spraying, the eggs were air-dried then transferred to clean glass vials. Twenty of *T. remus* parasitoids with a male: female ratio of 1:2, were then introduced into each of the glass vials containing sprayed FAW eggs to assess parasitism. The experiment was replicated four times and arranged in a complete randomized design (Chepkemoi et al., 2023). The bioassays were maintained at temperature of 25-27°C, 50-70% relative humidity, and 12-hour light: 12-hour dark photoperiod. After 24 hours, the sprayed eggs were transferred to clean vials and *T. remus* adults were provided with honey. The mortality rate of the parasitoids was recorded daily until all individuals died. Dead parasitoids were surface sterilized and placed in Petri dishes lined with damp sterilized filter paper to promote fungal growth on the surface of the cadaver. The mycosis rate was determined by observing fungal development on the surface of the cadavers. Mortality of the FAW eggs and parasitism rates were evaluated (Chepkemoi et al., 2023).

### 2.6 Data analysis

FAW egg and adult *T. remus* mortality data were corrected for natural mortality using Abbott’s formula (Abbott, 1925). The data’s normality was tested using Barlett’s test before being subjected to one‐way analyses of variance (ANOVA). The variances of the percentage mortalities were calculated using a binomial generalized linear model with logit link function (Atkinson, 2015). When the treatments showed significant differences (P < 0.05), the means were separated using the Student–Newman–Keuls (SNK) test et al., 20. The lethal time for 50% mortality (LT_50_) was analyzed by Generalized Linear Model (GLM), using the function ‘dose. p’ from the MASS library, to estimate the lethal time to 50% mortality (LT_50_) (Opisa et al., 2018). The parasitism rate was calculated as the percentage of the number of parasitoid adults relative to the total number of host eggs and then analyzed using ANOVA. Mycosis data were analyzed using one‐way ANOVA, while the sex ratios assessed using chi-squared test (χ2 test) (Chepkemoi et al., 2023). All data analyses were performed using R (version 3.2.5) statistical software packages (R Core Team, 2018).

## 3 Results

### 3.1 Direct effects of entomopathogenic fungal strains on *Telenomus remus* and FAW eggs

Significant differences were observed in mortality of *T. remus* adults among all the treatments seven days’ post-treatment (F = 10.16; df = 3, 12; P = 0.001) (**Table 2**). *Beauveria bassiana* ICIPE 621 recorded the lowest adult mortality, whereas *M. anisopliae* ICIPE 7 recorded the highest mortality (**Table 2**). The strains of *M. anisopliae* ICIPE 7 and ICIPE 78 were identified as the most virulent, with LT_50_ less than 2 days. In contrast, *M. anisopliae* ICIPE 41 and *B. bassiana* ICIPE 621 had LT_50_ values of 2.52 and 2.89 days, respectively (**Table 2**). Calculated LT_50_ values for the strains indicate that, overall, the *M. anisopliae* strains induced quicker mortality with lower LT_50_ values compared to *B. bassiana* strain. The percentage of mycosis of the cadavers ranged from 16.25 – 47.50% with significant differences observed among the strains (F = 6.306; df = 4, 15; P = 0.003) (**Table 2**). The fungal strains did not significantly impact FAW eggs hatchability after exposure to the infected *T. remus* adults seven days’ post-treatment (F = 5.487; df = 3, 12; P = 0.632) (**Table 2**).

**Table 2:** Percentage of mortality of *Telenomus remus* adults; LT_50_ and FAW eggs induced mortality by the different fungal strains after direct infection.

Fungal strains	*Mean* adult mortality (%)	FAW egg mortality (%)	Parasitism rates (%)	Female/Male (F: M)	*Telenomus remus* LT_50_ (Days)	Mycosis of *T. remus* cadavers (%)
ICIPE 7	81.40 ± 4.08a	44.25 ± 9.14a	42.50 ± 5.55b	2:1	1.00 ± 0.15	38.75 ± 10.5a
ICIPE 78	76.51 ± 5.69b	61.25 ± 8.43a	38.75 ± 8.43b	2:1	1.91 ± 0.07	40.00 ± 8.4a
ICIPE 41	67.97 ± 6.01b	39.75 ± 6.52a	40.25 ± 6.42b	2:1	2.52 ± 0.08	16.25 ± 6.25b
ICIPE 621 Control	61.39 **±** 6.50b	55.00 ± 5.99a	40.00 ± 4.56b 83.25 ± 5.94a	2:1	2.89 ± 0.09	47.50± 9.242a

Means within a column followed by the same letters are not significantly different by Student–Newman–Keuls test at P < 0.05.

Furthermore, the number of emerged *T. remus* adults was significantly influenced by fungal species infection and showed variability among the different strains (F = 9.357; df = 4, 15; P < 0.001) (**Table 2**). The control treatment had the highest parasitoid emergence rate at 83.25%, while the lowest was recorded in *M. anisopliae* ICIPE 78 at 38.75% (**Table 2**). The sex ratios of the emerged parasitoids were similar across all treatments and did not differ significantly (P > 0.05, χ^2^ test).

### 3.2 Indirect effects of entomopathogenic fungal strains on *Telenomus remus* adults

#### 3.2.1 Cumulative mortalities of *Telenomus remus* adults and FAW eggs after indirect exposure to FAW eggs infected with different strains concentrations

**Figure 1:**
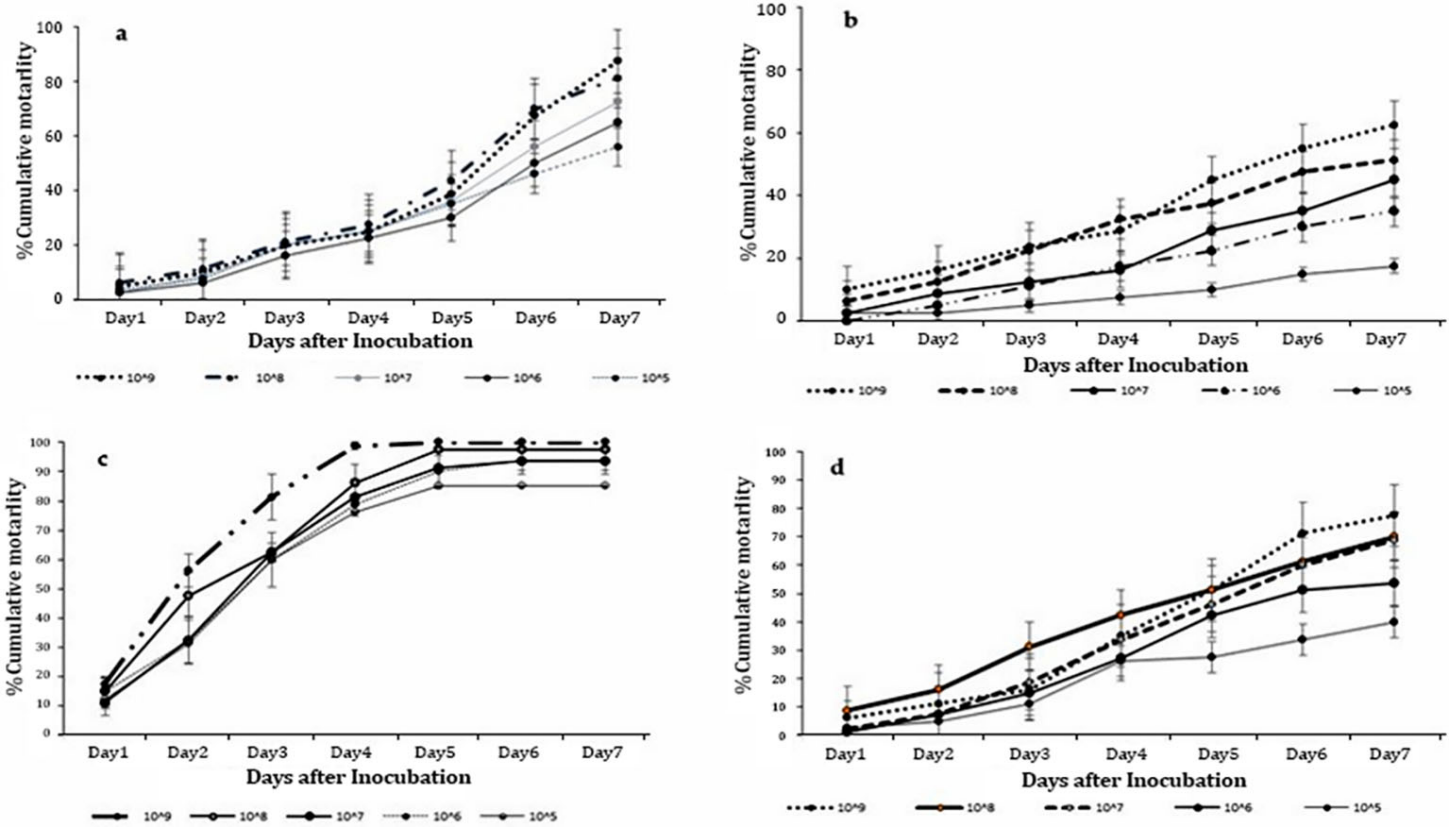
Cumulative mortalities of *Telenomus remus* adults indirectly exposed to *Metarhizium anisopliae s.l.* concentrations: **(A)** ICIPE 7, **(B)** ICIPE 41, **(C)** ICIPE 78, and **(D)** *Beauveria bassiana* ICIPE 621 *s.l*.

Significant differences in mortality of *T. remus* adults were evident/obtained in all the concentrations of *M. anisopliae* ICIPE 41 (F = 14.83; df = 4,15; P < 0.001), ICIPE 7 (F = 14.35; df = 4, 15; P < 0.001), ICIPE 78 (F = 16.34; df = 4,15; P < 0.001) and *B. bassiana* ICIPE 621 (F = 14.77; df = 4, 15; P < 0.001) strains, respectively, seven days’ post-treatment. Among all the strains, the highest concentration of 1 x 10^9^ conidia ml^-1^ recorded the highest *T. remus* adult mortality with ICIPE 41, ICIPE 7, ICIPE 78 and *B. bassiana* ICIPE 621 recording 62.50%, 87.50%, 100.00% and 77.50%, respectively (**Figure 1**). Also, significant differences in mycosed cadavers were noted among all the concentrations (F = 15.64; df = 4, 15; P < 0.001) with mycosis ranged from 18.75% to 78.75%.

Furthermore, significant differences in FAW egg mortality were observed among all the *M. anisopliae* concentrations of *M. anisopliae* ICIPE 41 (F = 2.77; df = 4, 15; P < 0.001), ICIPE 7 (F = 7.063; df = 4, 15; P < 0.001), and ICIPE 78 (F = 2.304; df = 4, 15; P < 0.001) strains, respectively, seven days post-treatment. In contrast, *B. bassiana* ICIPE 621, did not show significant effects across the various tested concentrations (F = 4.79; df = 4, 15; P = 0.3). Among all the strains, the highest concentration led to the highest FAW larval adult mortality, with ICIPE 41, ICIPE 7, and ICIPE 78 recording 26.25%, 49.25% and 51.25%, respectively (**Table 3**).

**Table 3:** Mean FAW egg mortality after infection with the different fungal isolates at the various concentrations.

Concentrations	*M. anisopliae* ICIPE 7 *s.l.*	*M. anisopliae* ICIPE 41 *s.l.*	*M. anisopliae* ICIPE 78 *s.l.*	*B. bassiana* ICIPE 621 *s.l.*
Mean FAW egg mortality (%)				
1 × 10^9^	49.25 ± 5.42a	26.25 ± 3.86a	51.25 ± 7.55a	30.75 ± 4.03a
1 × 10^8^	38.50 ± 5.98ab	21.75 ± 7.13ab	42.75 ± 7.85ab	25.25 ± 3.94a
1 × 10^7^	35.50 ± 2.40ab	20.25 ± 3.84ab	38.25 ± 6.81ab	27.5 ± 4.52a
1 × 10^6^	30.00 ± 5.96b	14.75 ± 2.84b	36.00 ± 3.87ab	22.75 ± 3.47a
1 × 10^5^	25.75 ± 2.50bc	15.25 ± 1.55b	32.25 ± 8.08b	21.00 ± 2.86a

Means within a column followed by the same letters are not significantly different by Student– Newman–Keuls test at P < 0.05.

#### 3.2.2 Effect of entomopathogenic fungi concentrations on parasitoid emergence

The average emergence of *T. remus* adults was not significantly impacted by *M. anisopliae* and *B. bassiana* strains, with ICIPE 41 (F = 1.415; df = 5, 18; P = 0.266), ICIPE 78 (F = 0.913; df = 5, 18; P = 0.495) and *B. bassiana* ICIPE 621 (F = 1.197; df = 5, 18; P = 0.35) showing no significant differences. However, ICIPE 7 showed a significant effect (F = 3.174; df = 5, 18; P = 0.0315). Additionally, the sex ratios of the F1 progeny of *T. remus* did not differ significantly across the different fungal strain treatments when compared to the controls (**Table 4**).

**Table 4:** Effects of the various fungal strain concentrations on the emergence of *Telenomus remus* (parasitism rates) in the laboratory (Mean ± SE (%)) and their female/male (F:M) proportion.

Concentration	*M. anisopliae* ICIPE 7 *s.l.*	*M. anisopliae* ICIPE 41 *s.l.*	*M. anisopliae* ICIPE 78 *s.l.*	*B. bassiana* ICIPE 621 *s.l.*
1 × 10^9^	40.25 ± 8.94b (2:1)	48.25 ± 5.71a (2:1)	48.75 ± 7.55a (2:1)	69.25 ± 4.03a (2:1)
1× 10^8^	48.00 ± 7.74b (2:1)	52.00 ± 9.96a (2:1)	57.25 ± 7.85a (2:1)	74.75 ± 3.94a (2:1)
1 × 10^7^	49.25 ± 6.49b (2:1)	60.25 ± 6.49a (2:1)	61.75 ± 6.81a (2:1)	72.5 ± 4.52a (2:1)
1 × 10^6^	67.50 ± 6.59ab (2:1)	63.25 ± 9.59a (2:1)	64.00 ± 3.87a (2:1)	71.5 ± 5.42a (2:1)
1 × 10^5^	69.25 ± 2.32ab (2:1)	66.00 ± 2.86a (2:1)	67.75 ± 8.08a (2:1)	75.25 ± 2.17a (2:1)
Control	72.75 ± 2.95a (2:1)	71.50 ± 6.41a (2:1)	70.25 ± 5.44a (2:1)	82.00 ± 3.29a (2:1)

Means within a column followed by the same letters are not significantly different by Student–Newman–Keuls test at P < 0.05.

## 4 Discussion

Our study showed that there was a direct effect of EPF on *T. remus*, causing adult mortality to *T. remus* when exposed to the different dry conidia of entomopathogenic fungi isolates. Upon direct application, *M. anisopliae* and *B. bassiana* strains exhibited pathogenicity towards *T. remus* adults with ICIPE 7 inducing the highest adult mortality while *B. bassiana* ICIPE 621 resulted in the least mortality rate. *Metarhizium anisopliae* strains have generally demonstrated higher virulence than *B. bassiana* strain in several studies. Previous reports by Martinez et al. (2020) recorded a lower parasitoid *Coptera haywardi* (Hymenoptera: Diapriidae) infection by *B. bassiana* strain through a direct application into the soil under semi-natural conditions, when both biological control agents were applied in combination to manage *Anastrepha obliqua* (Macquart) (Diptera: Tephritidae) fruit flies. The authors observed a significantly lower mortality of *A. obliqua* under semi-protected conditions as compared to the laboratory (Martinez et al., 2020). The mortality reduction could be attributed to the fact that environmental factors like temperature, solar radiation and humidity have been shown to diminish *B. bassiana* survival and potency (Robert et al., 2011). This low infection rate allows flexible adjustment of application periods for both parasitoid and fungus in the field, resulting in minimal or no parasitoid infection, as described with the use of *B. bassiana* and the parasitoid *Diadegma semiclausum* (Hellen) (Hymenoptera: Ichneumonidae) in controlling *Plutella xylostella* (L.) (Lepidoptera: Plutellidae) (Monnerat et al., 2002). Presa-Parra et al. (2021) similarly observed that direct infection of *M. anisopliae* resulted in significant 40% mortality rate in *Cephalonomia stephanoderis* Betrem (Hymenoptera: Bethylidae) adults, coffee berry borer (*Hypothenemus hampei*) parasitoid. Another study also recorded 90% mortality in *Diadegma semiclausum* (Hellen), a parasitoid of *P. xylostella*, after infection by the fungus *Zoophthora. radicans* (Abbas, 2020). However, other reports have also shown that *Monochamus alternatus* (Coleoptera: Cerambycidae), adult pine sawyers were successfully infected with *B. bassiana* at lethal dose simply by walking across fungus- contaminated non-woven fabric strips with a concentration of 3.5 x 10^8^ conidia cm -2 (Shimazu, 2004).

The strains also had varying killing speeds with the most virulent strains which recorded LT_50_ less than 2 days being *M. anisopliae* strains. Despite the fact that all entomopathogenic fungi have a similar infection mechanism, variation in virulence among strains could be due to factors such as proteases, lipases and chitinases, responsible for cuticle destruction and insect penetration (Ortiz-Urquiza and Keyhani, 2013). Previous research by Migiro et al. (2010) also found that entomopathogenic fungi from different species or strains within the same genera caused varying mortality rates in *Liriomyza huidobrensis* (Blanchard) (Diptera: Agromyzidae), a pea leafminer.

*Metarhizium anisopliae* ICIPE 78 recorded the highest conidial transfer from adult *T. remus* parasitoid to *S. frugiperda* eggs, followed by *B. bassiana* ICIPE 621. In contrast, *M. anisopliae* ICIPE 41 recorded the lowest FAW egg mortality rate, indicating less effective conidial transfer and fungal impact. The transfer of fungal conidia by a non-target insect, such as *T. remus* to the FAW eggs is known as entomopathogenic vectoring. During this process, *T. remus* parasitoid successfully transmitted some degree of the different fungal strains of the fungi to FAW eggs causing significant mortalities. In previous studies, *B. bassiana*, *B. brongniartii* and *M. anisopliae* were vectored by species of collembolans (*Fernaldella fimetaria*, (Lepidoptera: Geometridae), *Hestina assimilis* (Lepidoptera: Nymphalidae), and *Proisotoma minuta* (Collembola: Isotomidae), which ended up causing mortality on mealworms, *Tenebrio molitor* (L.) (Coleoptera: Tenebrionidae) (Dromph, 2003). This level of entomopathogenic fungi vectoring (entomovectoring) combined with parasitism showed positive results in the management of FAW as previously reported with *Cotesia icipe* (Hymenoptera: Braconidae) by Chepkemoi et al. (2023). In the current study, parasitism of *T. remus* on FAW eggs was highest in samples exposed in the controls, but no significant difference in parasitism was recorded among the different fungal strains. This parasitism extent of *T. remus* on FAW, unaffected by other external biopesticides was corroborated by a laboratory study conducted by Laminou et al. (2020), where it was reported that *T. remus* parasitized averagely 78% of FAW eggs, whereas *Trichogrammatoidea* sp. (Hymenoptera: Trichogrammatidae) parasitized only 25%. Additionally, *T. remus* was capable to parasitize FAW egg clusters, even when the eggs were completely covered with scales, whereas *Trichogrammatoidea* sp. could only parasitize eggs that were not covered. Upon parasitization of FAW eggs, *T. remus* emergence was the highest in controls as compared to those exposed to different fungal strain treatments.

To assess the most effective entomopathogenic fungal concentrations for inducing ovicidal effects on *S. frugiperda* eggs and their subsequent impact on *T. remus* adults, our results indicated a general reduction in the lethal efficacy of all the four strains, as the concentrations decreased from 1 × 10^9^ conidia ml-1 to 1 × 10^5^ conidia ml^-1^. The highest mortality rate of *S. frugiperda* eggs was observed in *M. anisopliae* ICIPE 78, followed by *M. anisopliae* ICIPE 7 and *B. bassiana* ICIPE 621, whereas least egg mortality was observed in *M. anisopliae* ICIPE 41. Our results collaborate with those described by Akutse et al. (2013) who showcased ovicidal effects on *S. frugiperda* after exposure to entomopathogenic fungi in a laboratory study using a single discriminating concentration (1 x 10^8^ conidia ml^-1^) of *M. anisopliae* ICIPE 41 and ICIPE 7 which caused 97.5% and 96% mortality rates of eggs and neonates, outperforming strains ICIPE 78, ICIPE 40 and ICIPE 20, which caused mortalities of 87.0%, 83.0% and 79.5%, respectively. A field trial of biocontrol-based IPM on FAW included two treatments: one spray of *Bacillus thuringiensis* (NBAIR-BT25) and another of *M. anisopliae* (NBAIR Ma-35) at 2.0 x108 cfu/g. The trials achieved ovicidal effects of 76% and 71.64% on egg masses, respectively (Varshney et al., 2021). The two treatments induced larval mortalities of 80% and 74.4%, respectively, over a period of 60 days’ after application (Varshney et al., 2021). Idrees et al. (2022) reported that *B. bassiana* strains, when applied at a concentration of 1 x 10^9^ conidia ml^-1^, resulted in a 96% mortality rate among 2^nd^ instar FAW larvae. Other studies by Anand and Tiwary (2009) showed that, both *B. bassiana* and *M. anisopliae* demonstrated ovicidal effects on other species of *Spodoptera* Guenée, *viz., S. litura* (Lepidoptera: Noctuoidae). Additionally, these fungi showed efficacy against other lepidopterans including *Phthorimaea operculella* Zeller (Lepidoptera: Gelechiidae) potato tuber moth, with Khorrami et al. (2018) reporting a 63% larval mortality.

Exposure of *T. remus* parasitoids to infected FAW eggs showed that higher EPF’s concentrations led to significant mortality among adult parasitoids compared to lower concentrations. *Beauveria bassiana ICIPE* 621 strain at the concentration of 1 x 10^9^ conidia ml^-1^, recorded the highest *T. remus* adult mortality rate, whereas lowest adult mortality was recorded in *M. anisopliae* ICIPE 78 at the concentration 1 x 10^5^ conidia ml^-1^. More research have shown that *M. anisopliae* has the ability to infect *Diglyphus isaea* Walker (Hymenoptera: Eulophidae), emphasizing the need for careful application, to prevent negative impacts on beneficial parasitoids (Migiro et al., 2010).

Emergence of *T. remus* F1 progeny was not affected by the different fungal strains showing that the parasitoid mortality occurred after parasitizing the FAW eggs. This is an indication that these strains could be effectively combined with *T. remus* parasitoid for FAW management. These findings also highlight that even though increased concentrations may increase parasitoid mortality, careful timing is crucial in such way that the females’ parasitoids will have parasitized the hosts before dying. Ferreira Agüero and Neves (2014) similarly observed that *Telenomus podisi* (Hymenoptera: Scelionidae) parasitism rates on *Euschistus heros* eggs, treated with *M. anisopliae* strain. Rashki et al. (2009) also found that releasing the parasitoids before applying entomopathogenic fungi resulted in reduced impact on the parasitoids, in aphid control (Hemiptera: Aphididae). Additionally, Abbas (2020) also evaluated the effect of *B. bassiana* on the emergence and longevity of *Aphidius matricariae* (Haliday), the peach aphid, *Myzus persicae* (Sulzer) parasitoid and reported that the longevity and sex ratios of mummies treated with fungus were similar to untreated ones, four days’ post treatment. Therefore, timing of application for both the parasitoid and the fungus plays a crucial role in their interactions. Some field research have also demonstrated that integrating low dose of EPF with multiple parasitoid species can be an effective pest management strategy (Abbas, 2020).

There was no significant effect on the sex ratios of the emerged *T. remus* adult parasitoids from both the direct and indirect fungal applications when compared to controls. Moreover, all the tested concentrations resulted in a predominance of female progeny in the F1 generation, which is crucial for parasitoid reproduction. Further research is thus necessary to investigate the lifespan and survival rates of the F1 progenies. Potrich et al. (2009) found no variations in the sex ratios of *Trichogramma pretiosum* emerging from *Ephestia kuehniella* (Lepidoptera: Pyralidae) eggs treated with *M. anisopliae* (Unioeste 22 strain), when compared to control. Furthermore, Polanczyk et al. (2010) reported that *M. anisopliae* had no effect on the sex ratios of *Trichogramma atopovirilia* (Hymenoptera: Trichogrammatidae) even when diet was contaminated and eggs immersed in conidial solutions. While studying the effects of *M. brunneum* on controlling *Spodoptera littoralis* (Boisduval) (Lepidoptera: Noctuidae) larvae (Miranda-Fuentes et al. 2020) and its interaction with the parasitoid *Hyposoter didymator* (Thunberg) (Hymenoptera: Ichneumonidae) in melon plants, Miranda-Fuentes et al. (2021) found that, high concentrations of the fungus decreased the longevity of the parasitoids, but did not affect the productive capacity of the females three days post- treatment.

## 5 Conclusions

Our findings indicated that the autodissemination process of the fungal inoculum, whether vectored by the FAW eggs to the parasitoids or from the parasitoids to the eggs, along with varying the fungal concentrations, could have detrimental effects on the parasitoids. The parasitoids infected with *M. anisopliae* strains exhibited a higher conidial transfer to their hosts, leading to increased FAW egg mortality. Moreover, results from indirect application indicated that conidia concentrations, which were sublethal to parasitized *S. frugiperda* eggs, did not adversely affect the performance and emergence of *T. remus* adults. This suggests that both control agents could be effectively used concurrently, with an appropriate time of release. To accurately assess compatibility, more field studies are required to provide enough information on how the two biocontrol agents perform in a non-controlled environment. Further research is needed to explore more effective application methods to enhance the impact of the two biocontrol agents in field conditions.

## Data Availability

The original contributions presented in the study are included in the article/supplementary material. Further inquiries can be directed to the corresponding author.
